# When Less Is More: Reduced Usefulness Training for the Learning of Anticipation Skill in Tennis

**DOI:** 10.1371/journal.pone.0079811

**Published:** 2013-11-11

**Authors:** Nicholas J. Smeeton, Raoul Huys, David M. Jacobs

**Affiliations:** 1 Faculty of Education and Sport, University of Brighton, Eastbourne, United Kingdom; 2 Institut de Neurosciences des Systèmes, Aix-Marseille Université, Marseille, France; 3 Inserm, UMR_S 1106, Marseille, France; 4 Centre National de la Recherche Scientifique, Universidad Autónoma de Madrid, Spain; 5 Facultad de Psicología, Universidad Autónoma de Madrid, Spain; University of California, Merced, United States of America

## Abstract

Participants in this study practiced with feedback to anticipate the left-right direction of forehand tennis shots played by stick-figure players. A technique based on principal component analysis was used to remove dynamical differences that are associated with shots to different directions. Different body regions of the stick-figure players were neutralized with this procedure in the pretests and posttests, and in the practice phases. Experiment 1 showed that training is effective if during practice information is consistently present in the whole body of the player, but not if the information is neutralized in the whole body in half of the practice trials. Experiment 2 showed that training is effective if the variance associated with the direction of the shots is consistently present in one body region but neutralized in others, and that transfer occurs from practice with information in one body region to performance in conditions with information preserved only in other regions. Experiment 3 showed that occlusion has a much larger detrimental effect on learning than the applied neutralization technique, and that transfer between body regions occurs also with occlusion. Discussed are theoretical implications for understanding how biological motion is perceived and possible applications in a type of training referred to as reduced usefulness training.

## Introduction

Humans are often remarkably skilled at perceiving another person's intentions from their movements. Being able to reliably perceive intentions and thereby anticipate the outcome of actions is important for skilled sports performance. In highly time-constrained discrete events that involve whole-body actions, as for instance typically found in fast ball sports, skillful individuals anticipate their opponents' actions, which aids their timely response. To explain anticipatory behavior, researchers have searched for detectable kinematic patterns that reveal the to-be-perceived intentions [1], [2] or have focused on forward models and other internal constructs that are hypothesized to underlie the capacity to anticipate [3], [4]. As indicated by the former approach, the detection of information that specifies the intentions, which are causally linked to the outcomes of the considered actions, is critical for anticipatory behavior. Therefore, if an individual wishes to become a skillful anticipator, (s)he must learn to attend to the movement patterns that reliably indicate the outcomes of those actions.

How can one facilitate the process of coming to attend to the more useful patterns of information? The prevailing training method in the anticipation skill literature is to augment the information that is presumably used. This is often done through verbal instructions that guide the learner to specific locations. An alternative method to enhance perceptual learning is referred to as *reduced usefulness training*. This method aims to stimulate learners to detect more useful informational variables by lessening the usefulness of the variables that are initially used by novice perceivers [5].

In the present study a novel type of reduced usefulness training is proposed and experimentally investigated by manipulating the information pertinent to specific body regions using a technique based on principal component analysis (PCA) [6]. Our results have theoretical implications for understanding how (human) movement is skillfully perceived and how this perceptual skill can be acquired.

Previous research has shown that directing attention sequentially to particular body regions, typically first proximal then distal to the major axis of rotation, can facilitate anticipation skill learning [7]. Directing attention to these body regions is best accomplished in a manner that maximizes active search and exploration from learners but minimizes their awareness about procedural and perceptual aspects of their performance [8], [9], [10], [11], [12], [13]. Instructions are thought to constrain the learners' search while they discover the relevant information themselves. In addition, although some strategic aspects such as location attended to may be accessible to consciousness and verbalizable, the procedural aspects derived from the strategy are not. Other researchers have abstained from providing verbal instructions and instead visually augment regions at temporally relevant moments [14], [15]. A potential limitation of instructional approaches, however, is that they may over-constrain the learner's exploration or may not constrain the exploration in a functional manner. One of the important challenges faced in this field therefore is to constraint practice sufficiently so that learners quickly converge onto the useful variables, without providing too many constraints and turning learning into a less effective explicit and verbalizable process.

In contrast to instructional approaches, a predominant focus in the Gibsonian perceptual learning literature is on candidate informational variables. Rather than focusing on potentially informative regions in the stimulation, methods such as correlation and regression analyses are used to determine which informational variables are good predictors of the to-be-perceived properties and which informational variables explain the variance in the behavior of observers. Knowledge about variable use forms the basis of the above-mentioned reduced usefulness training [16], [17] (cf. Experiment 2 of [18]). This type of training is not only concerned with identifying the relevant informational variables, but also with how individuals come to use them. Weaker informational variables that are typically used by novices are made even less useful in the set of training stimuli encountered during practice, while the usefulness of the variables that are typically used by experts remain unchanged. The continued use of the variables typically used by novices therefore results in learners being less successful, which, according to reasoning behind the method, promotes a quicker discovery of the stronger informational variables.

Jacobs and colleagues [5] investigated how to manipulate the usefulness of visual information to help learners discover the more useful information for the perception of the relative mass of two colliding balls. By purposefully selecting their sets of training stimuli, they reduced the informational value of lower-order kinematic variables that may be used for the task (i.e., the collision exit-speed difference, the scatter-angle difference, and their linear combination). Two methods to manipulate the usefulness of variables were applied. In the first one, referred to as the *zero-correlation method*, practice sets of collisions were selected in which the correlations between the considered lower-order variable(s) and the to-be-perceived property was zero (unlike in sets of collisions that are not purposefully selected). With this method, convergence on more useful variables was achieved most effectively by rendering the lower-order variable that was initially used by the novice learners (but not other variables) useless. Participants who initially relied on a variable that was not rendered useless in practice found that their performance stayed fairly consistent during training.

A second method to reduce the usefulness of candidate informational variables, referred to as the *no-variation method*, addresses the variance of the variables rather than the correlation of the variables with the to-be-perceived property. In one of the practice sets of collisions in [5], for instance, after each collision the two balls had the same exit speed. This means that an observer who relies exclusively on the variable exit-speed difference would perceive the two balls as being of the same mass on each trial, which makes the variable useless for the task at hand. The no-variation practice was less successful than the zero-correlation practice: Nullifying the variance of the initially-used informational variable (i.e., no-variation practice) rather than its relation with the to-be-perceived property (i.e., zero-correlation practice) resulted in participants falling back to using the variable again in the posttest when its usual variance was restored.

Reduced usefulness training has previously been used in tasks for which the informational variables, and novices' use of them, were known. In many studies concerning the skillful anticipation on the basis of whole-body movements, however, the informational variables are not known beforehand, and are difficult to identify due to the high-dimensionality of the information spaces involved, and limitations in the methods typically used. Typically, attempts to understand how skilled anticipators differ from novice counterparts have tended to use spatial and temporal occlusion methodologies. In these methods, certain body locations (e.g., hips) or time periods (e.g., 80 ms before ball-racket contact) are occluded in the (video) displays and the effect of the occlusion on performance is determined by comparing it to non-occluded control stimuli. Decrements in performance relative to the non-occluded control indicate that the occluded body region or time period provides information for anticipating the outcome of an event (see [19] for a critique). Regardless of the particular striking action or the particular sport used, novice performance is most impaired when distal regions (i.e., the end-effector linkage) are occluded. Skilled individuals also pick up information from more proximal regions (e.g., in cricket: [20], [21], [10], [22]). In agreement with the results from occlusion studies, eye movement studies generally show that novices' point of gaze is predominantly directed toward the end effector. Skilled anticipators search more systematically and fixate on regions such as those located close to the major axis of rotation (e.g., in tennis: [23]; in soccer: [24]). In other words, both occlusion and gaze-registration methods are geared toward identifying the body regions and temporal windows of an action that somehow contain the informational variables, but they do not and cannot identify the relevant information explicitly.

As already hinted at, in the case of many real-life situations, informational variables for anticipation of whole-body movements are difficult to identify. In part this is due to the large number of (mechanical) degrees of freedom. A given outcome can be achieved in numerous ways [25], [26], [27] so that, for some actions at least, a unique one-to-one mapping between local aspects of kinematic movement patterns and action outcomes may not exist. At the same time, one may assume that a dominant amount of the variance associated with a given action outcome is present in all actions. Huys and colleagues [6] aimed to identify this variance. They used PCAs to capture the time-evolving patterns underpinning tennis shots, and next investigated if these patterns are used for the anticipation of the shots. PCA aims to reduce high-dimensional datasets into lower-dimensional ones while minimizing information loss. A number of (orthogonal) principal components (sometimes referred to as modes) are identified and ranked via their associated variance. The presence of covariance in the data, as is typically the case in high-dimensional movement patterns, allows for a reduced description of the data. Consistencies in movement patterns across trials (i.e., covariance) show up in the first (few) components. Trial-to-trial variations, in contrast, co-vary less and are thus associated with components that capture less variance.

Huys and colleagues [6] found that the predominant patterns of (co-)variance in tennis shots to different directions had contributions from almost all body regions (albeit to a varying degree) and were not just associated with one or a few isolated body parts. Still, the largest contribution to the predominant modes came from the striking arm and racket region. The displacement of these regions contributed significantly to three distinct time-evolving patterns that captured approximately 90% of the variance in six tennis players' shot deliveries. Next, visual perception experiments showed that these three distinct patterns allowed for the accurate anticipation of shot direction (i.e., at the same level found when viewing the original shots). Huys and colleagues suggested that the detection of these patterns is a parsimonious way of extracting information about the whole-body action, and that global information pick-up may render anticipation robust against (local) trial-to-trial performance variations. Conversely, the piecemeal information pick-up from a single region may provide uncertainty given that whole-body actions can be (and are) performed in more than one way to achieve the same outcome. Evidence consistent with these ideas was reported in [19]. Relative to the discussion above, however, it still remains to be seen whether the PCA modes identified are the information in striking actions (in this context) or (merely) contain this information.

As an aside, although the notions of global and local information pick up have become in vogue over the last few years, they have never been defined (to our best knowledge). We (here) use these terms as relative ones and in the context of the existing literature: local, then, refers to a more or less single body region such as the racket, the shoulders, or the hips; global in our use designates multiple such regions and may (but need not) imply the whole body (plus tools).

Because of the difficulties in explicitly identifying informational variable(s) for the anticipation of human movement, the theoretical predictions derived from previous studies using reduced usefulness training for anticipation training are as yet unclear. As a result, it remains to be seen if some type of reduced usefulness training is applicable to tasks in which the informational variable(s) are unidentified. For example, how can one reduce the correlation between initially used variables and to-be-perceived properties if one does not know what the initially used variables are? This seems to indicate that, as long as the variables used by novices are not explicitly known, the zero-correlation method cannot be applied. However, if a novel type of reduced usefulness training can be developed that is effective when neither the specifying information for a given task nor the variables typically used by novices are known, then it could be used in a wide variety of real-life applications where the issue of informational complexity makes the explicit identification of these variables difficult or currently impossible.

As indicated above, in the case of anticipation in tennis, knowledge about the zones or body regions associated with successful anticipation for novices and experts is well documented and the low-dimensional pattern allowing for anticipation has been identified. In the present study we test if this knowledge is sufficient to successfully use reduced usefulness training. We examine whether reducing location-related information for determining shot direction in tennis with PCA techniques can be used to improve anticipation skill without explicitly knowing what that information is. Specifically, we examine whether removing information in the end effector, typically used by novice anticipators, can promote information pick up from additional regions, and hence lead to anticipation that has perceptual characteristics of skilled perceivers. Before we report our findings addressing this aim we report an experiment validating our training protocol and examining how reducing the usefulness of information from all body regions affects anticipation skill learning.

## Experiment 1

In this first experiment, we compared anticipation skill learning of three groups of participants. As in the subsequent experiments, participants judged the left-right direction of tennis shots (termed inside-out-cross-court respectively) played by stick-figure players. The first group, referred to as *reliable* group, viewed unmodified training stimuli during the acquisition phase of the experiment. In this practice situation information for anticipation was contained in all body regions [6]. The second group, referred to as *unreliable* group, viewed the same stimuli mixed with stimuli that had shot direction-specific differences eliminated from the shots (neutralized) in all body regions. By including neutralized stimuli in the stimulus set used in practice, all the informational variables that are contained in the movement patterns were made less reliable. The third group, referred to as *no practice* group, did not practice. The elimination of direction-specific differences was achieved with PCA techniques (see Methods section).

**Figure 1:**
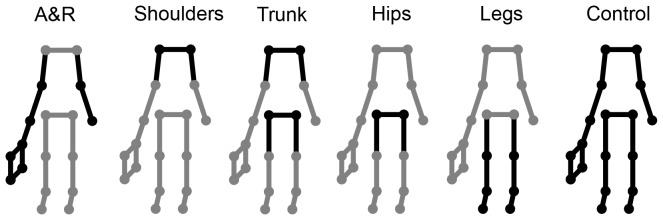
Static images representing body-region conditions used in pretests and posttests of all three experiments. Black dots indicate that direction-specific differences were preserved in the videos and gray dots indicate that these differences were neutralized.

Performance of the three groups was compared on pretests and posttests. During these tests, participants faced stimuli that contained direction-specific differences in specific body regions only. As shown in Figure 1, the used body regions were: arms and racket, shoulders, trunk, hips, legs, and whole body (i.e., control). These regions were chosen because several researchers have proposed them to be important for anticipation (e.g., [28], [29], [30], [6]). We expected that the reliable group would improve substantially with practice and, as a consequence, perform more successfully in the posttest than the unreliable group and the no practice group. Additionally, we expected the superior posttest-performance to be most pronounced when viewing stimuli with the direction-specific differences preserved in the proximal regions of the body, because these regions have been shown to be used by more skillful anticipators when anticipating movement outcomes [13], [23].

### Methods

#### Ethics Statement

All experimental and ethical approval procedures used in the three experiments were approved by the University of Brighton Faculty Ethics Committee. All participants gave informed written consent before participating in the study. In the case of participants under the age of 18 years old, written consent was obtained from a parent or guardian as well as verbal and written consent from the participant. Consent was documented via a signature on the consent form.

#### Participants

Thirty participants (20 male, 10 female) with a mean age of 16.4 years (*SD* = 7.7) were recruited for the study. Participants were randomly allocated to the reliable, unreliable, and no practice groups. None of the participants had substantial tennis playing experience.

#### Apparatus and stimulus production

The stick-figure simulations of tennis shots were presented to participants on a notebook computer (Acer, Aspire 5630, New Taipei city, Taiwan) using DMDX software [31]. Responses were registered with a Qwerty keyboard. The stimuli were constructed using MatLab (MatLab 6.5, MathWorks, Natick, MA). Each simulation was saved in audio-video interlaced format at a rate of 30 frames/s and lasted 1.8 s. The simulations started at the first backward movement of the right wrist from the ready stance and ended at the moment of ball-racket contact (no ball was visible throughout). The simulations were based on kinematic data collected and analyzed in [6]. In that study, retroflective markers were placed on 18 body and racket locations (left and right shoulder, elbow, wrist, hip, knee, ankle, and toe, and four racket positions) to record the kinematics of six right-handed tennis players as they performed forehand groundstrokes to four different target locations (forehand inside-out and cross-court shots to near and far targets). Inside-out and cross-court shots are defined here as forehand shots directed toward the left-hand or right-hand side of an opponent's court, respectively (from the perspective of the opponent). The recorded players were between 15 and 18 years of age and played tennis at the national level.

#### Neutralization

To create the simulations, the shots were analyzed and processed with a type of PCA that is applied to time series [6] (see [32] for a tutorial). In [6], the main PCA was run on a 5184-dimensional state vector *q_k_*(*t*): 18 (markers) ×3 (Cartesian coordinates per marker) ×4 (target locations) ×4 (shots [trials] per target location) ×6 (players)  = 5184 (dimensions). Conceptually, PCA consists of choosing a set of (orthogonal) vectors (*v^k^*) such that 
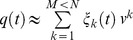
, where *M* is smaller than *N* (here *N* = 5184). This procedure, typically achieved via analysis of the covariance matrix of ***q*** (see [32]), provides *M* time-dependent coefficients, *ξ_k_*(*t*), associated with the vectors *v^k^*. In [6], more than 99.99% of the variance in the 5184-dimensional dataset was explained with the first 54 modes (principal components; i.e., for *M* = 54) of the PCA.

Our neutralization was based on these 54 modes *v^k^* identified in [6]. Each mode or eigenvector *v^k^* contains coefficients corresponding specific markers, Cartesian coordinates, target locations, trials, and players. Each coefficient can be interpreted as representing the degree of the contribution to mode *v^k^* of the marker associated with a Cartesian coordinate from a shot to a given target location, and from a given trial and given player contributed to the corresponding mode *k*. Shot-direction specific differences for a marker and Cartesian coordinate were neutralized by first averaging the coefficients corresponding to the left and right shot directions separately (i.e., averaging across player, trial and depth), and next averaging across the left-right shot directions, which for each mode *k*, results in a vector *v^k^* containing 54 coefficients (corresponding to 3 Cartesian coordinates ×18 markers). Similar neutralization procedures were performed in [6], [19] and [33]; more information can be found in those articles. In other words, the neutralization was achieved by averaging out inside-out (left) and cross-court (right) shot differences that are contained in the eigenvectors. By performing the averaging only for specific regions (corresponding to specific coefficients of the eigenvectors), shot-direction differences at those regions are eliminated while shot-direction differences at other regions are preserved. (Whenever shot-direction differences were averaged out for a given marker, it was done for all the Cartesian coordinates separately.) The manipulated (‘neutralized’) eigenvectors were then used to construct the simulation for a particular shot by multiplying the projection *ξ*(*t*) of each principal component *k* with the corresponding 54-dimensional vector *v^k^*, and summing (for each marker-Cartesian coordinate) the 54 resulting trajectories corresponding to the 54 modes. In [6], the PCA was performed on mean-subtracted and normalized (i.e., standard-deviation divided) time series. Therefore, to generate the simulations, each novel time series was multiplied by its corresponding standard deviation before adding its mean value. Player-specific standard deviations and means were used for the simulations of different players.

#### Test-phase stimuli

The stimuli for the pretests and posttests were identical to each other and for the three groups. Six conditions were used that differed according to the body and racket regions that the shot-direction differences in the dynamics were preserved in (Figure 1). Dynamic differences between shot directions in the remaining body or racket locations were neutralized (i.e., averaged out across shot directions). Sets of 12 stimuli were created for four players using the partly neutralized eigenvectors (i.e., one stimulus video per condition, shot direction, and player). Each stimulus was repeated 5 times. Hence, the pretest and posttest comprised 240 trials each (6 conditions [arm and racket, shoulders, trunk, hips, legs, control] ×2 shot directions [left, right] ×4 players ×5 repetitions). The presentation order in the test phases was randomized across condition, shot direction and player.

#### Acquisition-phase stimuli

**Figure 2:**
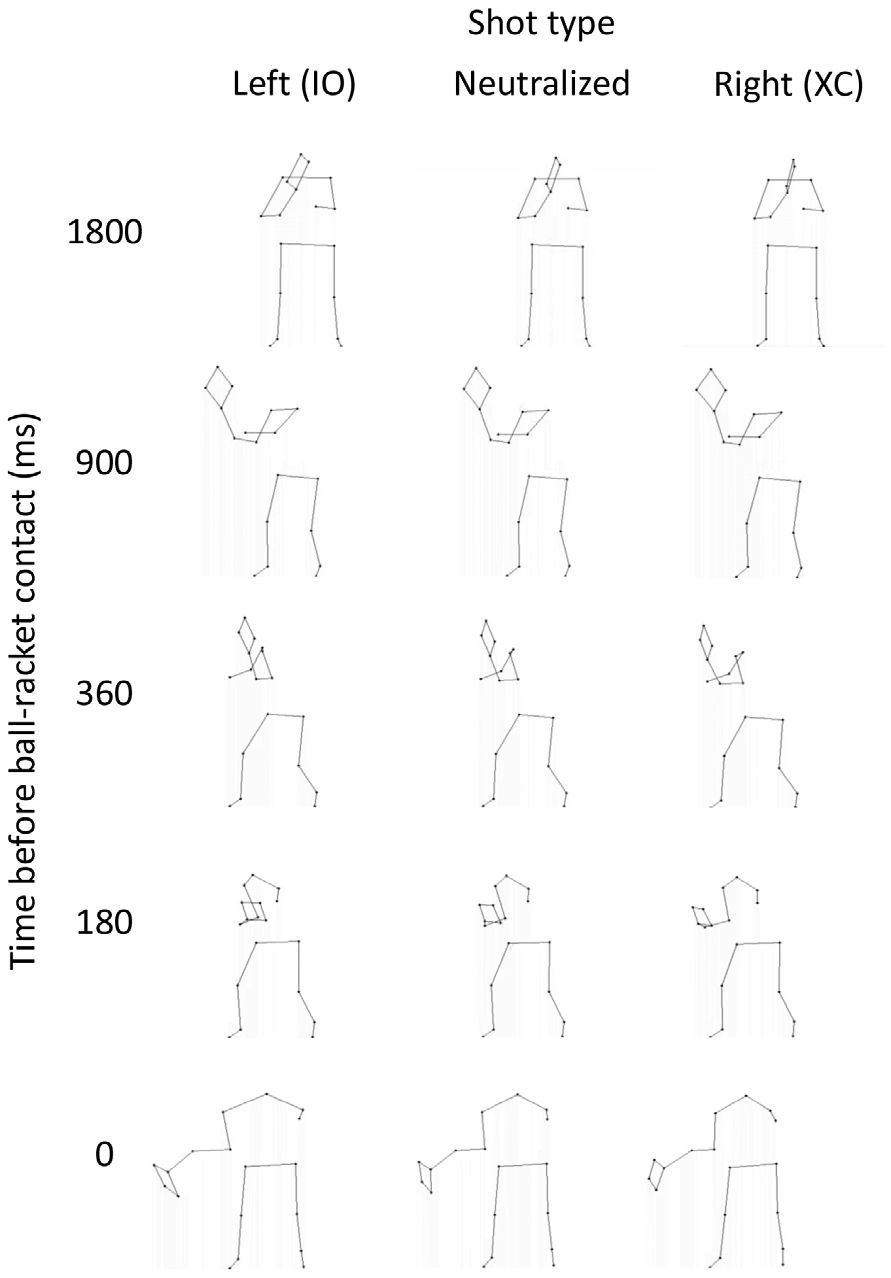
Experiment 1: acquisition-phase stimuli example frames at ms intervals from ball-racket contact for shots with dynamic differences preserved and neutralized. Shot to participants' left (left column), right (right column) and neutralized (center column). IO =  inside out, XC =  crosscourt.

The differences between the groups of the present experiment (and between our three experiments) concern the acquisition phases. For the reliable group, acquisition stimuli were used that preserved the dynamic differences between shot directions in all body regions. Figure 2 contains example frames from left and right shots where these dynamic differences are preserved and neutralized. Shots to the left and right were created for two players that were not used in the test phases, using the non-neutralized eigenvectors. These stimuli were repeated 15 times per block of trials. Four blocks of trials were used. The acquisition phase hence consisted of 240 trials: 4 blocks ×2 shot directions ×2 players ×15 repetitions. The order of presentation was randomized per block. For the unreliable group, the same acquisition stimuli were used, with the exception that on half of the trials all shot-specific differences were neutralized (via the procedure explained above). The no practice group did not receive any practice.

#### Procedure

Participants sat at a distance of approximately 0.5 m from the computer screen. They were informed that they would be shown forehand shots of stick figures ‘playing’ strokes to either their left or right hand side. They were tasked with determining the resultant shot direction by pressing the left or right hand shift key on the keyboard in all experimental phases: pretest, acquisition phase, and posttest. Before the pretest, participants were presented with an example shot from each test-phase condition for each shot direction (12 shots), presented in a block order. During the acquisition phase, the reliable and unreliable groups viewed their respective training videos. Participants in the reliable group were informed about the correct shot direction after each trial through a message that appeared on the screen after they gave their response. After giving their response, they watched a replay of that video. Participants in the unreliable group also received feedback after each acquisition trial, but feedback was genuine only for the 50% of the trials in which the genuine (non-neutralized) shots were shown. For the 50% of the trials that used the neutralized stimuli, these participants were given feedback indicating a shot to their left or right equally often, randomly allocated to the shots. An inter-trial interval of 3.5 s was used between all trials. The experiment took approximately 114 min to administer for the reliable and unreliable groups and approximately 54 min for the no practice group.

#### Data analysis

Anticipation accuracy was calculated as the percentage correct responses. The accuracy scores computed per individual and test phase were analyzed using a single two-way mixed design ANOVA with group (reliable, unreliable, no practice) as between-subjects variable and test phase (pretest, posttest) as within-subjects variable. In addition, the accuracy scores computed per individual, test phase, and body-region condition were analyzed using one-way ANOVAs with group as between-subjects variable. Significant effects of ANOVAs were followed up using Tukey's post hoc tests to locate differences between groups and Bonferroni-corrected dependent-samples *t*-tests to locate differences across the test phases for the groups. Effect sizes are reported as either partial eta squared (*η_p_^2^*) for main effects and interactions or Cohen's *r*. Assumptions of the ANOVAs were tested and corrected where appropriate.

### Results

#### Pretest to posttest

The ANOVA that concerned the pretest and posttest accuracy scores revealed a main effect of test phase, *F*(1,27) = 25.62, *p*<.01, *η_p_^2^* = .49, which was superseded by a significant Group × Test Phase interaction, *F*(1,27) = 14.71, *p*<.01, *η_p_^2^* = .52. There was a significant increase in accuracy scores for the reliable group, *t*(9) = 8.83, *p*<.001, *r* = 0.95, but no significant increase was found for the unreliable group *t*(9) = .46, *p*>.05, *r* = 0.15, or the control group, *t*(9) = .82, *p*>.05, *r* = 0.26. Tukey's HSD tests did not reveal differences between the groups in the pretest (*p*>.05). The mean pretest score was 62.3% (*SD* = 12.8). In the posttest, the reliable group (*M* = 85.4%, *SD* = 12.0) outperformed the unreliable (*M* = 65.2%, *SD* = 13.3) and the no practice groups (*M* = 67.8%, *SD* = 11.0). The difference between the latter two groups was not significant (*p*>.05).

#### Body regions

**Figure 3:**
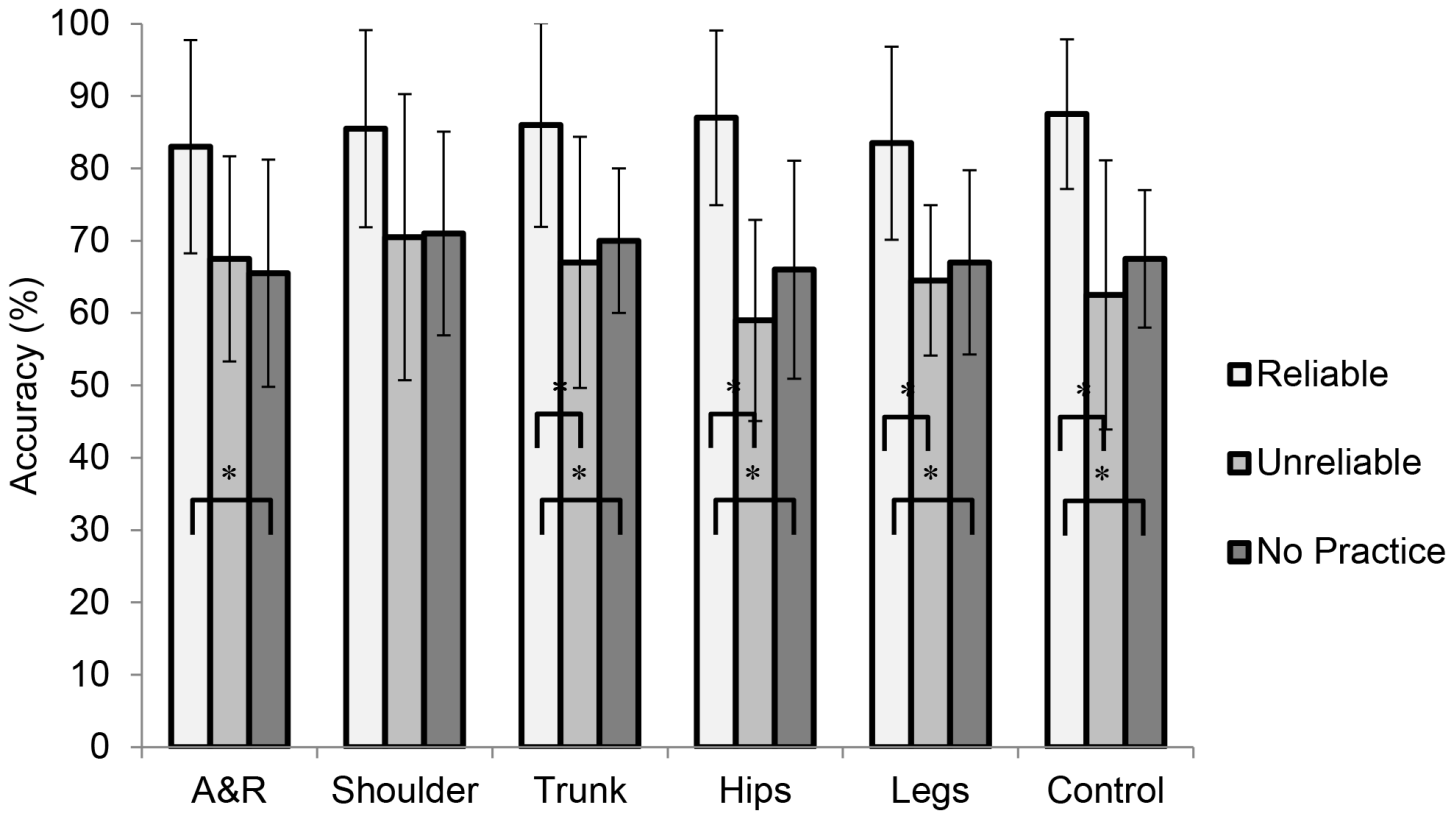
Anticipation accuracy scores (%) for the posttest of Experiment 1 for the reliable, unreliable, and the no practice groups. A&R =  Arm and Racket condition. Asterisks indicate significant differences between bracketed groups (*p*<.05).

The ANOVAs that concerned the anticipation accuracies computed per body-region condition did not yield significant results in the pretest (*p*>.05). The posttest results are illustrated in Figure 3. Main effects were observed for the following conditions: arms and racket, *F*(2,27) = 4.13, *p*<.05, *r* = 0.48; trunk, *F*(2,27) = 5.22, *p*<.05, *r* = 0.53; hips, *F*(2,27) = 11.26, *p*<.01, *r* = 0.67; legs, *F*(2,27) = 7.13, *p*<.01, *r* = 0.59; and control, *F*(2,27) = 9.67, *p*<.01, *r* = 0.65. In the shoulder condition there was only a tendency toward significance, *F*(2,27) = 2.81, *p*<.10, *r* = 0.41. For the arms and racket condition, Tukey's HSD tests revealed significant differences between the reliable and no practice groups (*p*<.05) but not between the reliable and unreliable group (*p*>.05). For the trunk, hips, legs, and control conditions, the reliable group performed significantly better than the unreliable and no practice group (*p*<.05), which did not differ significantly from each other (*p*>.05).

### Discussion

Overall, and as expected, the reliable group showed a large increase (*r* = 0.95) in performance from pretest to posttest. This can be interpreted as a validation of our experimental protocol. Furthermore, in the posttest, the reliable group performed better than the unreliable and no practice groups, demonstrating that a consistent presentation of information rather than a mere exposure to the stimuli leads to improvements in anticipation skill. The reliable practice was particularly effective when information was present only in proximal regions away from the end effector (i.e., trunk, hips, and legs); the posttest accuracy scores of the reliable group in these conditions were significantly higher than those for the other two groups. Additionally, the posttest accuracy scores for the reliable group were significantly higher than for the other two groups on the control condition.

These findings are consistent with the claim that practice with a set of stimuli in which shot-specific differences are consistently available in all regions allows learners to discover movement patterns that systematically co-vary with shot outcome. Removing shot-specific differences from 50% of the trials resulted in a failure to learn. When the sets of acquisition stimuli are considered as a whole, the manipulation reduces the co-variation of the shot outcome with all informational variables contained in the movement patterns. This includes the variables that are typically relied on by novices as well as the ones that are typically relied on by experts. Although reducing the usefulness of the variables that are typically used by novices may have been a useful first step to promote changes in variable use, the concurrent reduction of the usefulness of other variables probably frustrated the information search and left the corresponding participants without reliable alternatives.

## Experiment 2

In the first experiment learners improved their anticipation performance when direction-specific differences were consistently present in the whole body during the acquisition phase. No learning was observed when these differences were neutralized in 50% of the acquisition trials. In this second experiment, we tested whether learning occurs when direction-specific differences are consistently present in one part of the body while being consistently neutralized in the remaining parts. Because direction-specific differences in particular regions are consistently present, movement patterns in those regions will systematically co-vary with shot direction [6], [19]. This may allow learners to converge onto the use of such patterns. Our main hypothesis therefore is that learning will be observed in this experiment, independently of which region contains the (consistent) shot-specific differences.

The regions that were neutralized during acquisition were chosen on the basis of the typical performance of novice and expert anticipators. In one training group, the direction-specific differences were neutralized in the end-effector region (the right shoulder, arm, and racket linkage striking the ball), a region typically relied upon by novices (e.g., [22]). This group is referred to as the *body* group because the direction-specific differences were preserved for the body regions. In the other group, the *end-effector* group, the direction-specific differences were preserved for the end effector and neutralized for the other regions. Anticipation skill was assessed with the same pretests and posttests used in Experiment 1, containing conditions in which the direction-specific differences in specific body regions or in the whole body were present (Figure 1). As indicated, we expected both groups to improve their anticipation skill because information (in some region) was consistently available to both groups. However, predictions with regard to comparing the overall performance of the two groups are less clear. On the one hand, the end-effector group may improve more than the body group because the former group is trained on a region that contains more evident shot-specific differences than the latter [6]. On the other hand, reducing the usefulness of information in the end-effector region may promote the use of information from more proximal regions, and hence lead to performance that more closely resembles the performance of experts.

The experiment also raises questions about transfer. If learners converge onto the use of strictly local informational variables that are specific to the region which they are trained on, then transfer of learning from one region to others (e.g., from training with direction-specific differences in the end effector to posttest conditions with direction-specific differences in, say, the hips) is not expected. Hence, if transfer of learning is observed, then this would indicate that learning entails more than coming to attend to strictly local informational variables.

### Methods

The methods used for Experiment 2 were identical to the methods used for Experiment 1 with the following exceptions.

#### Participants

Twenty-two participants (8 male, 14 female) with a mean age of 21.0 years (*SD* = 2.4) were recruited. They were randomly allocated equally to the end*-*effector and body groups (no control group was used). None of the participants had substantial tennis playing experience.

#### Stimuli

**Figure 4:**
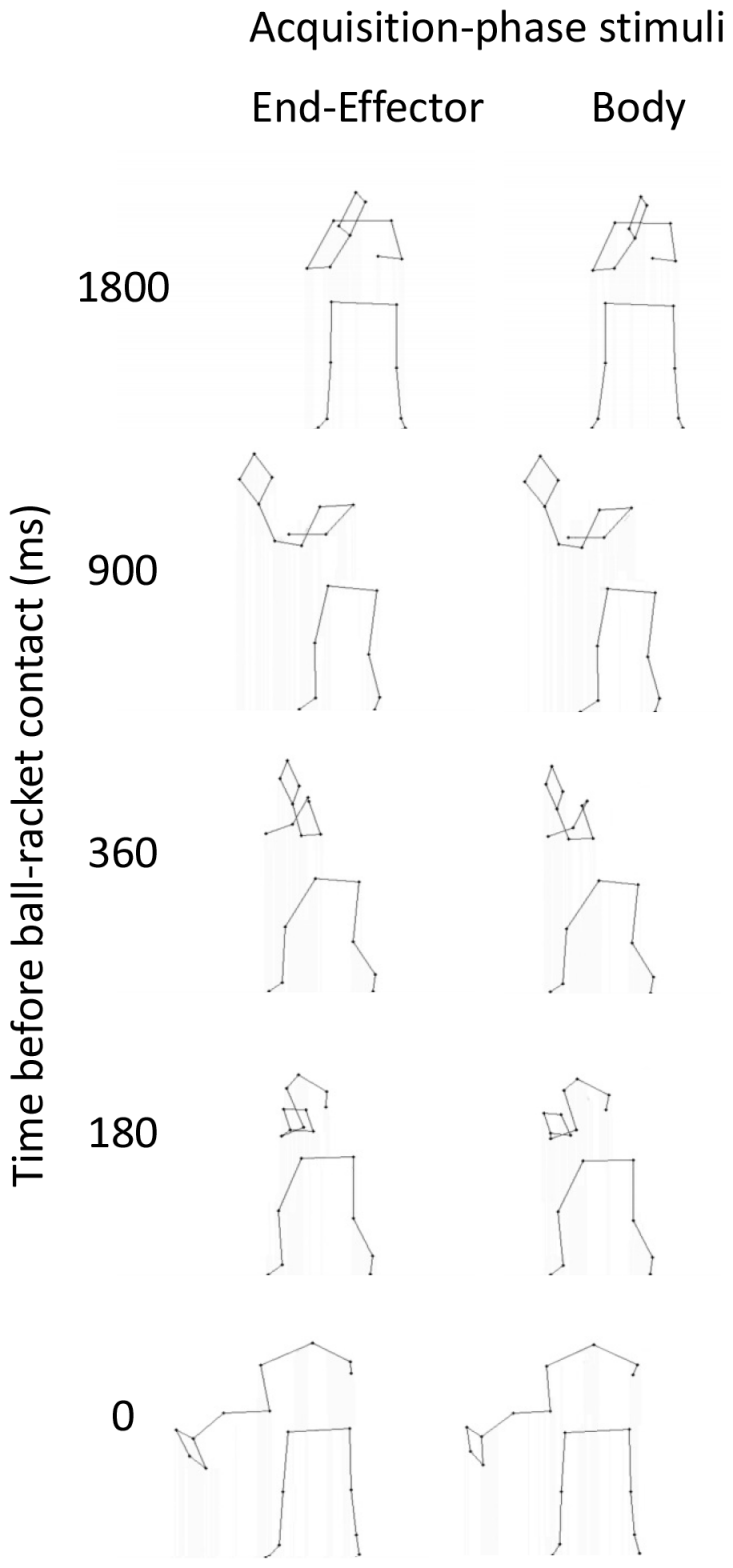
Experiment 2: acquisition-phase stimuli example frames at ms intervals from ball-racket contact. Left column depicts dynamic differences preserved in the end-effector only. Right column depicts dynamic differences preserved in the remaining body locations only. Both columns contain a shot to participants' left.

The end-effector and body groups differed from each other and from the groups in Experiment 1 only with regard to the acquisition stimuli. Example frames from the stimuli are presented in Figure 4. The stimuli created for the acquisition phase of the end-effector group preserved the direction-specific differences for seven markers: right shoulder, right elbow, right wrist, and 4 racket locations. The direction-specific differences for the other markers were neutralized with the procedure also used in Experiment 1. In the acquisition stimuli of the body group, the differences were preserved for all markers other than the seven arm and racket ones. Veridical feedback was given after the acquisition trials for both groups. In contrast to Experiments 1 and 3, the tennis players used to create the acquisition-phase stimuli were two of the four players that were also used to create the stimuli of the pretest and posttest. This was done to test whether stimulus familiarity was important. Because this was not found to be the case we did not further consider this difference.

#### Data analysis

The accuracy scores computed per individual and test phase were analyzed using a two-way mixed design ANOVA with group (end effector, body) as between-subjects variable and test phase (pretest, posttest) as within-subjects variable. Because this experiment contained only two groups, the anticipation accuracies computed per body-region condition were analyzed using two 2-way mixed design ANOVAs, one for the pretest and one for the posttest. For the latter ANOVAs, the between-subjects variable was group (end effector, body) and the within-subjects variable was body region (arms and racket, shoulders, trunk, hips, legs, and control).

### Results

#### Pretest to posttest

The ANOVA that concerned the pretest to posttest accuracy scores revealed a main effect of test phase, *F*(1,20) = 132.63, *p*<.001, *η_p_^2^* = .87. Both groups increased their mean shot prediction accuracy. For the end-effector group the pretest and posttest means were 61.4% and 86.3% (*SD* = 9.3 and *SD* = 2.5) and for the body group these means were 60.8% and 85.3% (*SD* = 10.8 and *SD* = 2.7). There was neither a significant effect of group nor a significant Group × Test Phase interaction (*p*>.05).

#### Body regions

**Figure 5:**
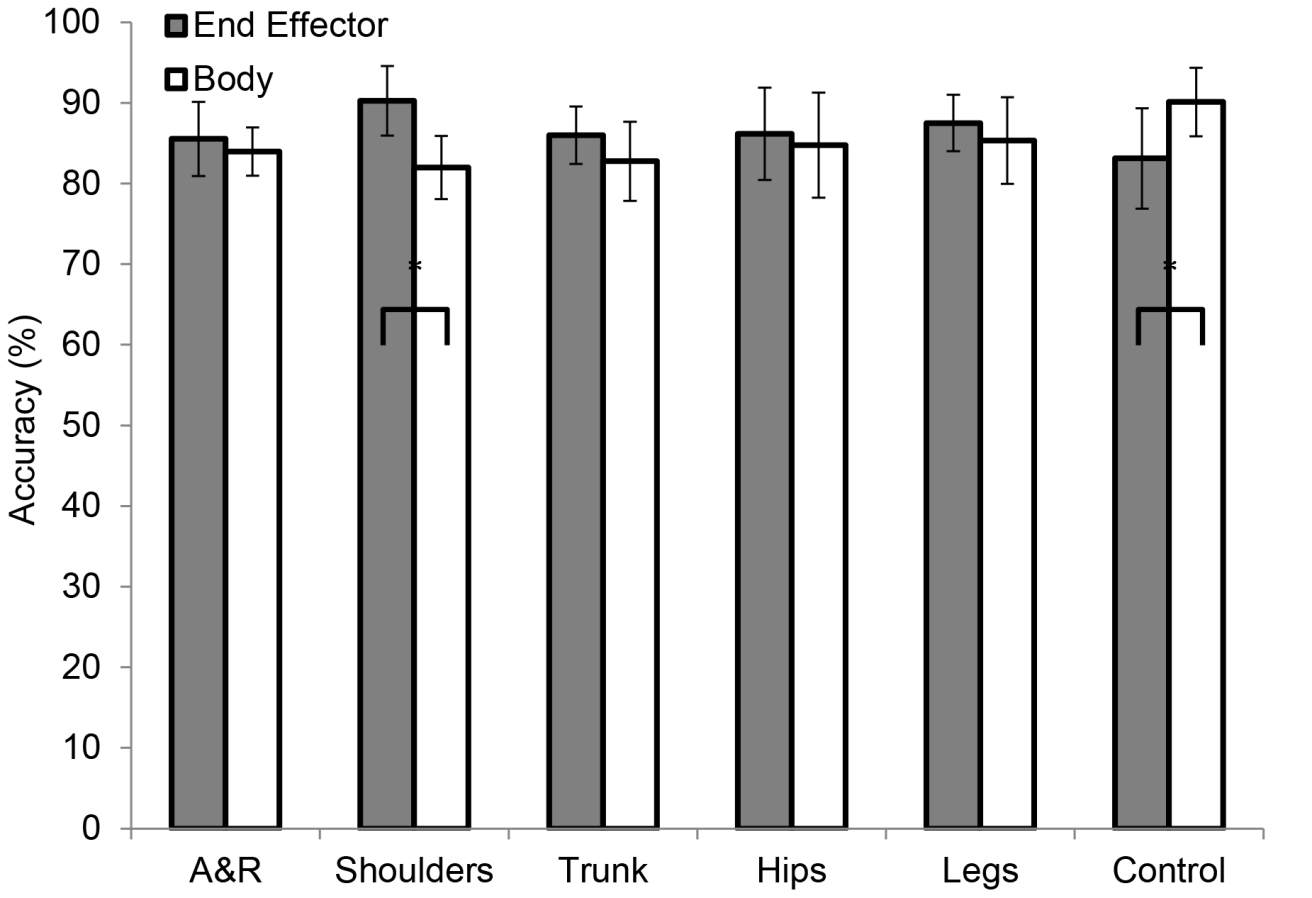
Anticipation accuracy scores (%) for the posttest of Experiment 2 for the end-effector and body groups. A&R =  Arms and Racket condition. Asterisks indicate significant differences between bracketed groups (*p*<.05).

In the pretest, the ANOVA on the anticipation accuracy per body region and group did not reveal significant effects (*p*>.05). In the posttest, there was a significant Group × Body Region interaction, *F*(5,100) = 7.30, *p*<.01, *η_p_^2^* = .27, but no main effects (*p*>.05). The posttest results are presented in Figure 5. To follow up the interaction, Bonferroni-corrected independent-samples *t*-tests were computed that compared the difference between the groups on each of the body-region conditions. A significant difference was observed in the control condition, *t*(20) = 3.11, *p*<.05, *r* = 0.57; the body group (*M* = 90.3%, *SD* = 4.2) was more accurate than the end-effector group (*M* = 83.2%, *SD* = 6.2). There was also a significant difference in the shoulder condition, *t*(20) = 4.78, *p*<.05, *r* = 0.73; here, the end-effector group (*M* = 90.0%, *SD* = 4.3) was more accurate than the body group (*M* = 81.6%, *SD* = 3.9). No other significant group-differences per region were observed (*p*>.05).

### Discussion

The training conditions of the end-effector and body group were both effective for anticipation skill learning. Together with the results of Experiment 1, this shows that for learning to be effective, information in at least one body region must be consistently present, but it does not have to be present in all body regions. We did not observe group differences in the overall posttest results. In the control condition of the posttest, however, there was a small performance advantage (*r* = 0.57) for the body group over the end-effector group. Because control-like (i.e., unmodified) events are the rule outside the laboratory, this result suggests that training without direction-specific differences in the end effector has more practical benefits than training without direction-specific differences in the rest of the body. These are encouraging results for the design of reduced usefulness training because they demonstrate (a) that training remains effective under neutralization, and (b) that neutralizing the region that is typically used by novices is more beneficial than neutralizing the rest of the body despite the fact that this region contains most of the shot-specific differences.

Both groups showed transfer of learning to stimuli that contained direction-specific differences only in body regions that they were not exposed to in the acquisition phase. The end-effector group increased performance from 57.3% to 85.9% when information was present in the arms and racket region (as trained on), but also increased their accuracy from 64.4% to 86.7% on average when information was available only in the body regions that did not feature in their training. This effect was mirrored in the body group. Participants in the body group increased their accuracy from 62.3% to 83.7% on average when information was present in regions that featured in their training, and from 60.3% to 83.9% when information was contained in the arms and racket region (not trained on). These effects are unlikely to be explained by general increases in perceptual sensitivity or familiarity to the tennis stimuli, because no improvements were found in the unreliable group of Experiment 1 that experienced the same exposure rates to the stimuli. The next experiment further addresses the observed transfer.

## Experiment 3

In Experiment 2 we observed transfer of learning to body regions that did not contain direction-specific differences during the acquisition phase. This finding implies that learning does not only consist of a convergence onto the use of strictly local and region-specific informational variables. Still, our work is based on the well-documented assumption that learning entails a change in variable use. How, then, can we understand the transfer? Body parts do not move in isolation. Instead, especially during expert performance, the neuromuscular apparatus forms temporal task-specific linkages, referred to as synergies or coordinative structures (e.g., [25], [26]). Perhaps because of these linkages, dynamical patterns may co-occur in different (local) body regions [6], [19]. For actions in which this is the case, learners may develop sensitivity to dynamical information through their experience with one region, and this sensitivity may transfer to other regions because those other regions carry the same region-independent information.

According to this reasoning, learning and transfer are predicted to occur when body regions are occluded instead of neutralized, though to a lesser degree. In this third experiment an occlusion protocol was used to test this idea. Two (new) groups of participants practiced the anticipation of shot direction with stimuli in which the body regions that were neutralized in Experiment 2 were occluded from view (i.e., not shown). Apart from using occlusion instead of neutralization the same experimental design was used. If the transfer observed in Experiment 2 was due to the use of region-independent dynamical patterns detected through attending to local regions, then transfer beyond the training stimulus is predicted for both groups. Also, if learning entails coming to use region-independent patterns, then one does not expect a strong decrement in performance with occlusion as compared to neutralization. The experiment can also be interpreted as a validation of the neutralization procedure. If less learning occurs in the present experiment as compared to Experiment 2, then neutralization techniques have advantages over occlusion (cf. [19]).

### Methods

**Figure 6:**
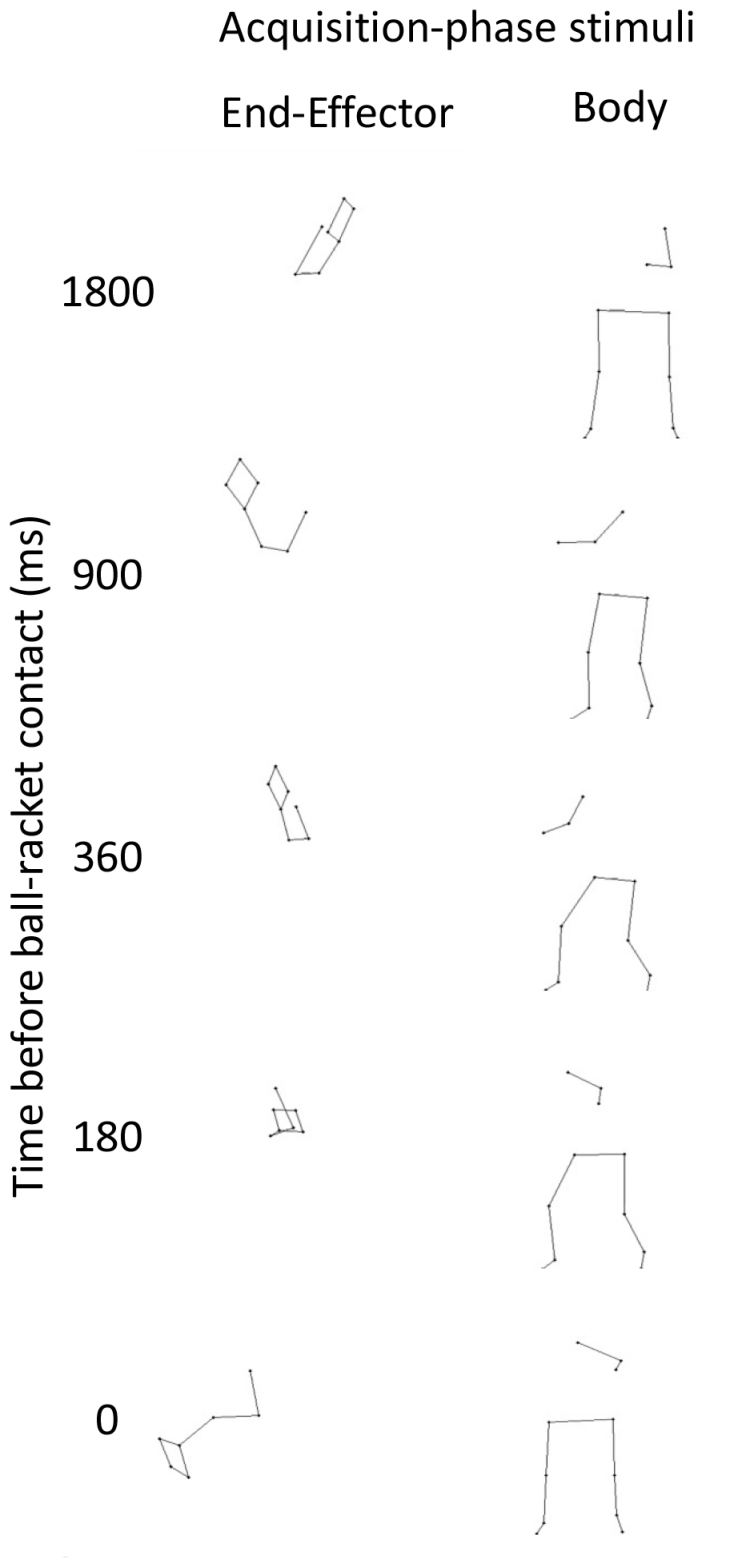
Experiment 3: acquisition-phase stimuli example frames at ms intervals from ball-racket contact. Left column depicts dynamic differences present in the end-effector only. Right column depicts dynamic differences present in the remaining body locations only. Both columns contain a shot to participants' left.

Thirty-eight new participants of mixed gender (19 male, 19 female) with a mean age of 19.5 years (*SD* = 1.1) were recruited for the experiment. They were allocated randomly to the end effector (*n* = 18) and body group (*n* = 20). Participants did not have substantial experience playing tennis. In the acquisition phase, body markers that were neutralized for the end effector and body groups of Experiment 2 were not shown for the corresponding groups of Experiment 3 (neither were the ‘sticks’ that joined the occluded markers). Example frames from the acquisition-phase stimuli are presented in Figure 6. The acquisition phase stimuli were created with the shots of two tennis players that were not used in the pretest and posttest. Otherwise the methods of Experiments 2 and 3 were identical.

### Results

#### Pretest to posttest

The analysis of the pretest to posttest accuracy scores per group revealed a main effect of test phase, *F*(1,36) = 12.76, *p*<.01, *η_p_^2^* = .26, but no significant main effect of group, *F*(1,36) = 1.48, *p*>.05, *η_p_^2^* = .04. These effects were superseded by a significant Group × Test Phase interaction, *F*(1,36) = 13.42, *p*<.001, *η_p_^2^* = .27. The interaction resulted from a significant increase in mean overall accuracy for the end-effector group from 59.1% (*SD* = 10.5) to 74.3% (*SD* = 15.6), *p*<.001, *r* = 0.76, while the body group did not improve: *M*_pretest_ = 62.5% (*SD* = 10.5), *M*_posttest_ = 62.3% (*SD* = 13.3), *p*>.05, *r* = 0.02.

#### Body regions

**Figure 7:**
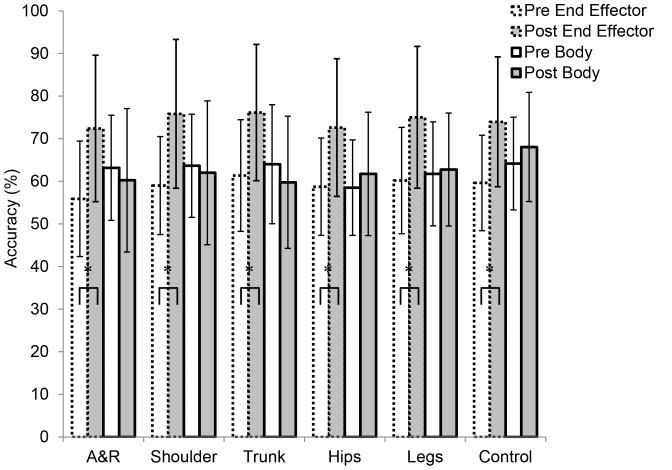
Anticipation accuracy scores (%) for the *end-effector* and *body* groups for the pretest (Pre) and posttest (Post) of Experiment 3. A&R =  Arms and Racket condition. Asterisks indicate significant differences between bracketed groups (*p*<.05).

The results per body region are summarized in Figure 7. For the end-effector group, performance significantly increased for all body-region conditions (*p*<.01). Notably, in all test conditions the effect sizes (*r*) for improvements were above the *large* benchmark (0.50; Cohen, 1977): 0.75 (arms and racket), 0.73 (shoulders), 0.72 (trunk), 0.67 (hips), 0.65 (legs), and 0.68 (control). In contrast, the body group did not significantly increase their performance in any condition (*p*>.05). Their effect sizes for increases in performance all fell below the *medium* benchmark (0.30): 0.17 (hips), 0.05 (legs), and 0.27 (control); as did two of three of the effect sized for decreases in performance: −0.17 (arms and racket), −0.08 (shoulders), and −0.35 (trunk).

### Discussion

In this occlusion experiment, performance of the end-effector group improved with practice. On average, their accuracy improved by 15.2%. This indicates that when the movement of the end-effector region is shown in isolation, information about shot direction is provided. In addition, the improvement transferred from the end-effector region to regions not visible during acquisition, lending support to the hypothesis that learners come to attend to dynamical patterns that are independent of body region. However, no improvement in accuracy was observed for the body group (−0.2% mean change). The effectiveness of reduced usefulness training by occluding body regions is therefore dependent on the regions occluded. The reason for this result is most probably related to the previously-reported finding that the end-effector region makes a larger contribution to the shot-direction specific dynamics than other regions do [6]. Consequently, the region-independent information may be easier to discover from the end-effector region.

For this result to be fully consistent with the hypothesis of locally detectable yet region-independent information the levels of performance in the posttests of Experiments 2 and 3 should have been similar. This was not the case. The overall posttest accuracy of the end-effector groups of Experiments 2 and 3 were 86.3% and 74.3%, respectively, and for the body group these scores were 85.3% and 62.3%. Hence, showing more body regions aids learning even if the additional body regions do not provide direction-specific information. This indicates that neutralization procedures have substantial advantages over occlusion procedures, which is consistent with results reported in [19].

## General Discussion

Learning partly involves converging on more useful informational variables. The process of convergence can be modified by manipulating the usefulness of candidate variables in the particular conditions that are encountered in practice. For instance, individuals typically do not change the variable used if the initially-used variables are reliable enough to maintain a satisfactory level of performance during practice, and they sometimes change more quickly if the initially-used variables are made less useful during practice [5]. The present study investigated the dependence of the learning process on the usefulness of informational variables in the context of anticipation of human movement. In contrast to the previous variable-usefulness studies, informational variables for the anticipation of tennis shots have not (yet) been explicitly identified. To manipulate the usefulness of variables, we manipulated the direction-specific part of the variance in the shots in specific regions of the action system (body plus racket), using PCA techniques similar to the ones used in [6] and [19].

Experiment 1 compared anticipation skill learning when movements were not manipulated, preserving all direction-specific differences and hence the usefulness of all informational variables, to a practice condition in which the usefulness of all informational variables was reduced. This comparison differs from the ones in previous studies (e.g., the zero-correlation practice in [5]). In previous studies, although initially used informational variables were made less useful, one of the informational variables always remained specific to the to-be-perceived property. Our Experiment 1 showed that reducing the usefulness of all informational variables obstructs learning. A possible explanation of this finding is that the usefulness of both the informational variables that individuals would normally converge toward and the initially used variables was reduced. Apparently, learners are not likely to change to use a variable with a reduced usefulness, even if the usefulness of the initially used variables is also reduced. (Alternatively, learners did change the variable(s) used, but settled on a variable(s) that did not improve their performance.) This implies that in order to achieve successful practice conditions one needs to consider how manipulations maximize the difference in usefulness of initially used variables and the variables that learners should ideally converge toward.

In Experiment 2, the informational content of tennis players' movements was neutralized (i.e., shot-direction specific differences were eliminated) in a region-by-region fashion. Either the end effector or the ensemble of regions other than the end effector was neutralized. Under these training conditions participants learnt. In addition, they showed transfer of learning to body regions in which information was not present during training. The observed transfer rules out the explanation that learning is limited to coming to attend to strictly local informational variables. One explanation of the observed transfer holds that learners come to rely on dynamical patterns that can be detected from local regions, even though the patterns themselves are (to some extent) region-independent. Recall, as demonstrated in [6], that the complex kinematics distributed across the action system associated with tennis shots (to different directions) can be partitioned into a few dominant co-varying patterns. By and large all body regions contribute to these patterns, although to different extent. That is, all (or most) body regions contain the same co-varying patterns (albeit to varying degrees). This means that practice with shot-specific differences in a particular region might help participants become sensitive to region-independent dynamics, which can also be detected through other body regions and can hence explain the observed transfer.

The procedure followed in Experiment 3 was similar to the one in Experiment 2 with one important exception; namely, in Experiment 3 direction-specific differences were eliminated in part of the training stimuli by occluding (i.e., not showing) the regions, whereas in Experiment 2 these differences were neutralized. Note that in the neutralization procedure shot-direction differences are eliminated (at particular markers) but the main part of the variance, which is related to the overall structure of the shots rather than to the shot specific differences, is maintained. Under occlusion, all the variance (associated with the particular markers) is omitted. Consequently, the occlusion method disturbs the patterns contained in tennis shots to a higher degree than the neutralization method (which has a different impact for different regions, as quantified in [19]). The applied partial occlusion preserved locally-defined as well as region-independent variables, and removed, or at least substantially impaired, variables defined across body regions (global variables). Learning and transfer of learning was observed for the end-effector group of Experiment 3, which is consistent with the claim that the end-effector region contains the most evident information for anticipation (e.g. [6]). The transfer indicates that learners are able to develop sensitivity to region-independent variables when these variables are presented to them in the end-effector region.

These findings are of interest to perceptual learning theory. For example, ecologically-motivated learning theories hold that observers come to detect higher-order informational variables (e.g., [34]). Higher-order variables are thought to be so because (a) they can be difficult for scientists to describe, (b) they may extend over time, and (c) they may extend over substantial spatial intervals (i.e., be global). For anticipation in tennis and potentially for the perception of human movement, our results add to this list. The higher-order variables may be region independent (cf. [6], [19]). The results thus imply that, rather than requiring exposure to a complete stimulus (i.e., whole body movement), ‘higher-order’ information may to some extent, be extractable from incomplete stimulus. This effect was dependent on the body regions missing from the stimulus, because no learning was observed for participants who practiced with stimuli in which the end-effector was region occluded.

One should note, however, that there were large differences in the results of Experiment 2 (neutralization) and Experiment 3 (occlusion). With occlusion less learning occurred than with neutralization: Performance increased by 25.0% on average in Experiment 2 and by 7.1% on average in Experiment 3. Moreover, in Experiment 3 no learning was observed for participants who practiced with stimuli containing an occluded end-effector. These differences suggests that globally defined informational variables are important, because these variables were available in the practice phase of Experiment 2 but not in the practice phase of Experiment 3. The differences also suggests that, from a practical perspective, training anticipation skill using neutralization methods should be favored over training with occlusion methods, because the former allow learners to rely on globally defined variables as well as on region-independent dynamical variables, hence leading to higher rates of success.

We find it worthwhile to note, speculatively, that the use of global variables may provide an alternative explanation for part of the transfer observed in Experiment 2. For the sake of the argument, consider the possibility that observers in the end-effector group of that experiment came to rely on the dynamics of the racket relative to the dynamics of the hips. This is a global variable because it is defined with markers from multiple body regions. Even though the hips did not contribute shot-specific differences in the acquisition phase, they still contributed a large proportion of their usual variance, implying that the racket dynamics relative to the hip dynamics may have been more useful for anticipation than the racket dynamics alone. Assume then, that an observer comes to attend to such a global variable during the acquisition phase. That observer may show better-than-chance performance in the posttest condition with shot-specific differences only in the hip region because the used global variable now contains shot-specific differences in the hips. This example illustrates that global variables may to some extent be useful during acquisition as well as during the different posttest conditions, allowing one to understand why, on the one hand, learners come to attend to the global variables during acquisition, and why, on the other hand, the use of these variables results in transfer.

It is also interesting to note a possible similarity between the occlusion and neutralization techniques considered in the present study and the no-variation and zero-correlation techniques used in [5]. In the no-variation practice of [5], candidate variables were rendered useless by removing their variance (i.e., by keeping them constant). Learners who initially relied on such variables could not use the variables during practice. Although these learners were temporarily forced to use other variables during practice, they were not confronted with the fact that the initially used variables were not the most useful ones, and they tended to fall back to their old strategies in a posttest where the initially used variables varied again. The zero-correlation practice maintained the variance in the variables but reduced their correlation with the to-be-perceived property. Learners in the zero-correlation groups could use the initially used variables during practice and hence experienced that these variables did not lead to satisfactory performance. These learners abandoned the variables and did not fall back to the initially used strategies in the posttest. It may be the case that the superiority of neutralization over occlusion techniques is partly related to a drawback of occlusion techniques - similar to one of the no-variation techniques: Although the occlusion temporarily forces learners to rely on other variables, learners may not learn that the initially used variables are not useful.

To summarize, we found support for the claim that the relative usefulness of informational variables during practice affects the learning process, and that reduced usefulness training can be used to learn to anticipate human movement. The finding that reducing all informational variables nullifies learning (Experiment 1) is new to the here-considered body of work about changes in variable use. The finding that selectively reducing informational variables is most effective if one reduces the informational variables that are typically used by novices (i.e., variables related to the end-effector region; Experiment 2) is consistent with previous findings, but extends them because they can be applied successfully without identifying the informational variables typically used by novices. The PCA methodology can be used in this context to reduce the usefulness of all informational variables in particular body parts (Experiment 2), which leads to different results than simply occluding these parts (Experiment 3). Results from Experiments 2 and 3 both demonstrate transfer of learning beyond the training stimulus and indicate that learning to anticipate human movement involves picking up (to some extent) globally-defined as well as region-independent information.
